# Subgenual cingulate resting regional cerebral blood flow in premenstrual dysphoric disorder: differential regulation by ovarian steroids and preliminary evidence for an association with expression of ESC/E(Z) complex genes

**DOI:** 10.1038/s41398-021-01328-4

**Published:** 2021-04-08

**Authors:** Shau-Ming Wei, Erica B. Baller, Pedro E. Martinez, Allison C. Goff, Howard J. Li, Philip D. Kohn, J. Shane Kippenhan, Steven J. Soldin, David R. Rubinow, David Goldman, Peter J. Schmidt, Karen F. Berman

**Affiliations:** 1 Section on Integrative Neuroimaging, Clinical and Translational Neuroscience Branch, NIMH IRP, NIH, Bethesda, MD USA; 2 Behavioral Endocrinology Branch; NIMH IRP, NIH, Bethesda, MD USA; 3 Laboratory of Neurogenetics, NIAAA, Bethesda, MD USA; 4 Department of Laboratory Medicine, NIH Clinical Center, Bethesda, MD USA; 5 Department of Psychiatry, University of North Carolina, Chapel Hill, NC USA

**Keywords:** Neuroscience, Psychiatric disorders

## Abstract

Substantial evidence suggests that circulating ovarian steroids modulate behavior differently in women with PMDD than in those without this condition. However, hormonal state-related abnormalities of neural functioning in PMDD remain to be better characterized. In addition, while altered neural function in PMDD likely co-exists with alterations in intrinsic cellular function, such a relationship has not been explored. Here, we investigated the effects of ovarian steroids on basal, resting regional cerebral blood flow (rCBF) in PMDD, and, in an exploratory analysis, we tested whether the rCBF findings were linked to the expression of ESC/E(Z) genes, which form an essential ovarian steroid-regulated gene-silencing complex. Resting rCBF was measured with oxygen-15 water PET (189 PET sessions in 43 healthy women and 20 women with PMDD) during three self-as-own-control conditions: GnRH agonist (Lupron)-induced ovarian suppression, estradiol add-back, and progesterone add-back. ESC/E(Z) gene expression data were obtained from RNA-sequencing of lymphoblastoid cell lines performed in a previous study and were examined in relation to hormone-induced changes in rCBF. In the rCBF PET data, there was a significant diagnosis-by-hormone interaction in the subgenual cingulate (*P*_FDR_ = 0.05), an important neuroanatomical hub for regulating affective state. Whereas control women showed no hormonally-related changes in resting rCBF, those with PMDD showed decreased resting rCBF during both estradiol (*P* = 0.02) and progesterone (*P* = 0.0002) add-back conditions. In addition, in PMDD, ESC/E(Z) gene expression correlated with the change in resting rCBF between Lupron-alone and progesterone conditions (Pearson *r* = −0.807, *P* = 0.016). This work offers a formulation of PMDD that integrates behavioral, neural circuit, and cellular mechanisms, and may provide new targets for future therapeutic interventions.

## Introduction

Premenstrual dysphoric disorder (PMDD), a prevalent and debilitating condition now included in the fifth edition of the DSM, affects approximately 5% of women of reproductive age and is characterized by the appearance of mood and behavioral symptoms confined to the luteal (post-ovulatory/premenstrual) phase of the menstrual cycle^1–3^. There is substantial evidence that circulating ovarian steroids modulate behavior differently in women with PMDD than in those without this disorder^4^. Indeed, in prior studies, approximately 55–70% of women with PMDD experienced remission of symptoms under conditions of gonadotropin-releasing hormone (GnRH) agonist-induced ovarian suppression, whereas PMDD symptoms recurred when physiological doses of ovarian hormones were added back during ovarian suppression^5,6^. In contrast, asymptomatic controls undergoing identical hormone manipulations experienced no changes in mood^5^.

Several neuroimaging studies have suggested a neurofunctional basis for hormonally-related behavioral phenomena (for review see refs. ^7,8^). In PMDD, the luteal (symptomatic) phase of the menstrual cycle has been associated with altered task-related activations in the medial and lateral orbitofrontal cortex (OFC), amygdala^9^, dorsolateral prefrontal cortex (DLPFC)^10^, insula and medial prefrontal cortex^11,12^. In addition to these state-related (i.e., menstrual cycle phase-specific) alterations, neurostructural and neurofunctional differences in women with PMDD have been reported that are independent of natural or pharmacologically-induced hormone fluctuations, and, therefore, are also independent of the presence of PMDD symptoms: in resting-state functional connectivity^13^; hippocampal/parahippocampal structure^14^; anterior cingulate, mOFC^15^, and DLPFC task-related activation^16^. Together, these observations suggest that there is an underlying trait-like neural circuit vulnerability in PMDD that likely co-exists with hormonal state-related abnormalities of neural functioning in PMDD and that may reflect changes in intrinsic cellular function.

In concert with this formulation, evidence of pathophysiology at the cellular level has also been reported. We recently found mRNA expression differences in lymphoblastoid cell lines (LCLs) from women with PMDD that could underlie the observed ovarian steroid-triggered behavioral sensitivity in this condition. Untreated (i.e., in steroid-free media) LCLs from women with PMDD showed significantly increased expression of a majority of the 13 gene members of the ESC/E(Z) complex^17^, an essential ovarian steroid-regulated gene-silencing complex^18^. However, the relationship between previously reported diagnosis-specific and hormonally-triggered neural circuit function and these cellular alterations in gene expression is unknown.

Here, to further investigate the neural substrates associated with the differential behavioral and cellular response in PMDD, we carried out a total of 189 PET scanning sessions in 63 women (43 healthy controls and 20 women with PMDD). Each woman was scanned under three different hormonal conditions during a six-month hormone manipulation protocol with a self-as-own control design. We chose the gold standard ^15^O-oxygen water positron emission tomography (PET) technique for measuring resting regional cerebral blood flow (rCBF), a parameter tightly coupled to local cerebral glucose metabolic rate^19^. This method thus provides a “metabolic” snapshot of brain function in a given brain state, including basal function during rest; rCBF is measured and directly mapped without reference to any other brain state and without the signal loss and susceptibility artifacts that occur in regions adjacent to bone and sinuses (e.g., inferior frontal regions) when MRI approaches are applied. While changes in resting-state functional connectivity in PMDD have been reported using fMRI, there has been no study of resting rCBF determined with PET. In addition, in an exploratory analysis of a subset of the women studied with PET, we searched for possible cellular concomitants of the rCBF findings by examining the relationship between gene expression in the ESC/E(Z) gene complex (a set of 13 genes involved in epigenetic silencing by H3K27 methylation that is regulated by ovarian steroids and the expression of which differs in lymphoblastoid cell lines from women with PMDD and control women^17^), and resting rCBF in specified brain regions (i.e., those differentially modulated by hormones and by a diagnosis of PMDD).

## Materials and methods

### Participant selection

Regularly-menstruating (i.e., 21–35 days in length) women who were medication-free, not medically ill (as assessed by history, physical exam, neurological exam, MRI, gynecological exam, pap smear within the last year, laboratory tests, and ECG), and not pregnant were recruited for the study. They were paid for their participation according to NIH volunteer guidelines. The study protocol was approved by the NIH CNS Institutional Review Board and Radiation Safety Committee, and all women provided written consent.

Participants with PMDD confirmed the timing and severity of their mood-related symptoms prospectively with daily self-ratings for three months prior to study entry using a four-item Visual Analog Scale^20–22^. For this study, the diagnostic criteria for PMDD were defined as a 30% increase in average negative mood (relative to the range of the scale used by each woman) during the week before menses compared with the week after menses, a more stringent criterion than that of DSM-4 or DSM-5. PMDD functional impairment was assessed by self-reports of distress and functional impairment on the Daily Rating Form ([DRF]^23^). The DRF criteria for functional impairment were as follows: a DRF score of 2 (minimal) or higher on one of four questions related to functional impairment (i.e., stayed at home or avoided social activities, had conflicts or problems with people, symptoms interfered with relationships at work or home, or symptoms interfered with work productivity) in at least three out of seven days pre-menses. Women who had significant negative mood symptoms on the DRF that occurred during the follicular phase of the menstrual cycle were excluded. DRF ratings and the results of both a semi-structured interview and a self-report questionnaire (i.e., the Menstrual Screening Questionnaire and the Menstrual Assessment Form, respectively) were employed to confirm that all women met the required number of symptoms specified in the DSM criteria for PMDD. The rating forms included an expanded version of the Visual Analog Scale used during the three-month baseline screening phase and a modification of the DRF^23^, both completed each evening. The ratings on both the DRF and the Visual Analog Scale assessed the severity of common symptoms of PMDD. These ratings were employed to confirm that each woman with PMDD met DSM-5 criteria for PMDD, and to measure symptom severity in all participants during the hormone-manipulation study. Finally, women with PMDD were excluded if they met the criteria for a current Axis I psychiatric diagnosis or any diagnoses within the past two years according to the Structured Clinical Interview for DSM-IV (SCID^24^).

Healthy controls were recruited through advertisements. Controls had no history of menstrual-related mood or behavior disturbances, as confirmed during the two months prior to entering the study by the same daily self-ratings used by the patients, and no current or past Axis I diagnosis, including alcohol and substance abuse, as confirmed by the SCID.

### Study design: hormone-manipulation protocol

**Fig. 1:**
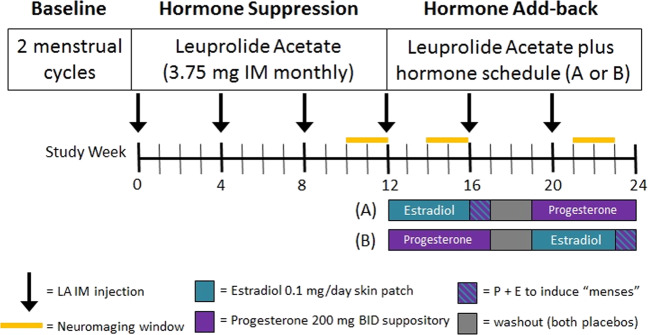
Schematic diagram of GnRH agonist-induced Hypogonadism and Gonadal Steroid Replacement. Following a two-month baseline evaluation period, women received 3.75 mg of Lupron (leuprolide acetate, purchased from TAP Pharmaceuticals, Chicago, IL) by intramuscular injection every four weeks for six months. Lupron alone was administered for the first 12 weeks. After the Lupron-alone period, women received, in addition to Lupron, 17β estradiol (0.1 mg/day) by skin patch or progesterone suppositories (200 mg BID) for five weeks each. Women then were crossed–over to the alternative treatment (in a double-blind, counterbalanced design). During the fifth week of estradiol add-back, progesterone suppositories (200 mg twice daily) were added to provide progesterone withdrawal-induced shedding of the endometrium and menses in order to prevent prolonged exposure of the endometrium to unopposed estrogen. The two replacement regimens were separated by a two-week washout period. Three PET sessions were acquired: during Lupron alone, estradiol add-back, and progesterone add-back periods. This experimental paradigm allowed us to compare brain function in the absence of ovarian steroids, as well as during the separate administration of estradiol or progesterone.

Throughout the six-month protocol, each participant received monthly injections of the GnRH agonist depot leuprolide acetate (Lupron, 3.75 mg IM), which suppresses the ovarian secretion of estradiol and progesterone. For the first three months of the protocol, women received only Lupron. At each clinic visit and before each scanning session, the Rating Scale for Premenstrual Tension Syndrome ([PMTS]^25^) was administered to each woman. For women with PMDD, responders to Lupron were defined by the remission of PMDD symptoms during ovarian suppression (i.e., PMTS scores <5) and by the absence of symptom cyclicity on the DRF^6^. Women with PMDD who did not meet symptom response criteria during Lupron-induced ovarian suppression were not continued on to the hormone add-back stage of the study. Those who did meet symptom response criteria during this initial Lupron-alone phase entered a three-month hormone add-back phase under double-blind, cross-over conditions while continuing to receive monthly Lupron injections (Fig. 1).

At the beginning of the add-back phase, women were randomly assigned to first receive five weeks of either transdermal 17B-estradiol 0.1 mg/day or progesterone vaginal suppositories 200 mg twice daily, with a two-week washout prior to a cross-over to five weeks of the second hormone add-back (i.e., progesterone in those first receiving estradiol or estradiol in those first receiving progesterone). Placebo suppositories and patches were used to maintain the blind. During the fifth week of estradiol add-back, all women received both estradiol and progesterone to induce menses. Plasma estradiol (liquid chromatography/mass spectrometry) and progesterone (RIA) levels were obtained at each study visit and before each imaging session (Table 2).

PMDD symptom-rating forms were completed daily by all women prior to study entry and during the six-month hormonal manipulation protocol (Table 2, Fig. 1).

### Acquisition and preprocessing of PET rCBF scans

The oxygen-15 water PET method was used to measure resting rCBF during each of three different hormone conditions: ovarian suppression during Lupron alone, Lupron plus estradiol-replacement, and Lupron plus progesterone-replacement (Fig. 1). Scanning took place during weeks 10–12 of the Lupron-alone condition (when ovarian steroid secretion is suppressed and, in women with PMDD, symptoms are typically in remission) and during the third or fourth week of each hormone-replacement condition (when women with PMDD [but not controls] are at risk for a recurrence of PMDD symptoms). During each hormone condition, two 60-s rCBF measurements (10 mCi H_2_^15^O/scan) were independently collected 6 min apart with a GE-Advance PET scanner in 3D mode (4.25 mm slice separation, 35 slices, the axial field of view 15.3 cm). Participants were instructed to lie still and to keep their eyes open during each resting scan.

Scans were corrected for background counts and attenuation (via a transmission scan) and were reconstructed into 32 axial planes (6.5 mm full-width-at-half-maximum). With SPM5 (Wellcome Department of Cognitive Neurology), the reconstructed PET data were anatomically normalized to an average template (across all women at all hormone conditions), scaled proportionally to remove variations in global blood flow, and smoothed using a 10 mm Gaussian kernel. The voxel-level data for the two rest scans for each hormone condition were averaged and entered into second-level analyses.

### Analyses of PET resting rCBF data

The averaged rest scans for each woman per hormone condition were entered as a repeated measure, and the diagnosis was entered as a between-groups measure in a factorial model in SPM5 with age and race as covariates of no interest. *F*-tests were performed to examine the main effects of both hormone conditions (i.e., Lupron alone, estradiol-replacement, progesterone-replacement) and diagnosis (PMDD, controls). Voxelwise findings were reported at a threshold of *P* ≤ 0.05, false discovery rate (FDR)-corrected, cluster threshold = 50.

To address the primary goal of the study, to search for brain regions where hormonal effects differed between patient and control groups, we performed an analysis of the interaction between diagnosis and hormone condition within a binary mask of the main effect of hormone condition across all participants. This approach was employed to restrict our primary analysis of interest (the interaction between diagnosis and hormone condition) to regions modulated by ovarian hormones. Results of the interaction analysis were further explored with post-hoc analyses across diagnoses and between hormone conditions of rCBF extracted from the peak voxel in clusters significant at *P* < 0.05, FDR-corrected. Finally, in women with PMDD, we examined the results of the rCBF interaction analysis as a function of symptom severity (i.e., PMTS scores) and of estradiol and progesterone plasma levels during the three separate hormone conditions using Pearson correlations in SPSS.

### Exploratory analysis of correlations between resting rCBF and ESC/E(Z) gene expression

In an exploratory analysis, we examined the relationship between hormone-induced changes in rCBF and ESC/E(Z) gene expression in a subset of healthy controls and women with PMDD who had participated in both the present PET study and a previous LCL RNA-sequencing (RNA-seq) experiment that investigated cellular function in women with PMDD (see^17^ for sample and experimental details). For the RNA-seq measures, normalized expression values (reads per kilobase of the transcript, per million mapped reads) of ESC/E(Z) genes were entered as variables for principal component analysis. Principal components were calculated in R using the prcomp function and visualized using the ggplot2 package^26^.

For brain regions where rCBF showed a significant diagnosis-by-hormone interaction at *P*_FDR_ ≤ 0.05, we correlated the first principal component of each woman’s ESC/E(Z) gene expression with her change in rCBF between Lupron alone and estradiol, as well as between Lupron alone and progesterone. Analyses were carried out separately for healthy control women and those with PMDD by generating Pearson correlations using SPSS.

## Results

Twenty women with PMDD and 43 asymptomatic controls participated in the study. There were no between-group differences in age, racial distribution, handedness, or years of education (Table 1). Ten of the 20 women with PMDD (50%) and 21 of the 43 asymptomatic controls (49%) underwent estradiol add-back first followed by progesterone add-back. Five women with PMDD had a past history of major depression, and one woman with PMDD met the criteria for past substance abuse disorder (alcohol).

**Table 1:** Demographics.

	Controls	PMDD	Statistical significance
*n*	43	20	
Age (years, mean ± SD)	33.9 ± 8.2	37.6 ± 8.3	*t*(61) = 1.8; *P* = 0.07, ns
Race	27C/13 AA/3A	9C/11AA	Fisher’s exact test, ns
Handedness	38R/5L (88.4%R)	20R (100%R)	*X*^*2*^ = 0.00005, ns
Years of education	16.1 ± 2.5	16.1 ± 1.9	*t*(61) = 1.4; *P* = 0.88, ns

### Hormone levels and behavioral findings

Plasma measurements of estradiol and progesterone confirmed ovarian suppression by Lupron, as well as replacement of the appropriate ovarian steroid during each add-back condition. There were no significant differences between controls and women with PMDD in hormone levels during any of the three conditions.

Women with PMDD showed symptom remission as measured by PMTS scales during Lupron alone, as well as recurrence of typical PMDD symptoms while on either estradiol or progesterone-replacement compared with the Lupron-only condition (Table 2). In contrast, controls experienced no mood or behavioral symptoms during any of the three hormone conditions. Finally, compared with controls, women with PMDD showed no difference in symptoms during the Lupron-only condition, whereas during estradiol and progesterone replacement, women with PMDD had significantly greater symptoms than controls.

**Table 2:** Premenstrual tension syndrome (PMTS) ratings and plasma hormone levels [mean ± SD].

	Lupron		Progesterone		Estradiol		ANOVA-R main effect of hormone condition F (*P* value)	ANOVA-R main effect of diagnosis F (*P* value)	ANOVA-R interaction of hormone-by-diagnosis F (*P* value)
	PMDD	Controls	PMDD	Controls	PMDD	Controls			
PMTS-rater	2.6 ± 3.7	1.3 ± 1.9	6.9 ± 7.5	1.1 ± 1.8	6.3 ± 5.3	1.1 ± 1.5	6.5 (0.002)	37.5 (<0.001)	7.5 (0.001)
Plasma Estradiol (pg/ml)	10.0 ± 5.7	11.8 ± 11.0	7.7 ± 4.7	9.4 ± 9.8	145.5 ± 106.2	123.5 ± 80.7	101.2 (<0.001)	0.5 (0.4)	0.4 (0.6)
Plasma Progesterone (ng/ml)	0.4 ± 0.2	0.3 ± 0.2	11.6 ± 4.7	13.3 ± 5.5	0.4 ± 0.2	0.4 ± 0.7	242.5 (<0.001)	0.6 (0.4)	0.3 (0.7)

*ANOVA-R* analysis of variance repeated-measures, *PMTS-rater* Premenstrual tension syndrome scale administered by clinician, *Est* estradiol, *Lup* lupron, *Prog* progesterone.

Post-hoc Bonferroni *t*-tests:

PMTS_Rater: PMD Est vs. Lup, *P* = 0.01; PMD Lup vs. Prog, *P* = 0.04; Est vs Prog, *P* = 0.6; Controls: all comparisons *P* ≥ 0.5

PMTS_Rater: Control vs. PMDD Est, *P* < 0.01; Control vs. PMDD Prog, *P* < 0.01, Control vs. PMDD Lup, *P* = NS.

Post-hoc testing compared estradiol levels of both PMDD and Controls during each hormone condition

PMDD: Est vs Lup, *P* = 0.00001; Lup vs Prog, *P* = 0.1; Est vs Prog, *P* = 0.00001

Controls: Est vs Lup, *P* = 1.7E-10; Lup vs Prog, *P* = 0.02; Est vs Prog, *P* = 5.3E-11

Controls vs PMDD: Est, *P* = 0.37; Lup, *P* = 0.49; Prog, *P* = 0.46.

Post-hoc testing compared progesterone levels of both PMDD and Controls during each hormone condition

PMDD Est vs Lup, *P* = 0.04; Lup vs Prog, *P* = 1.3E-8; Est vs Prog, *P* = 9.6E-8

Controls Est vs Lup, *P* = 0.3; Lup vs Prog, *P* = 8E-18; Est vs Prog, *P* = 1.2E-17

Controls vs PMDD: Est, *P* = 0.13; Lup, *P* = 0.36; Prog, *P* = 0.26.

PMTS ratings and blood samples were collected on the day of the scan. Blood samples were centrifuged, aliquoted, and stored at −70 °C until the time of assay. Plasma levels of progesterone were analyzed by radioimmunoassay (Diagnostic Systems Laboratory, Webster, TX). Intra-assay and inter-assay coefficients of variation for progesterone were 7.0–7.3% and 8.0–9.2%, respectively. Because plasma levels of estradiol during both the Lupron alone and progesterone add-back conditions were anticipated to be at the lower limits of detectability for standard RIA, estradiol was assayed by liquid chromatography/mass spectrometry^40^.

### Main effects of diagnosis and of hormone condition on resting rCBF

Whole-brain voxel-wise analysis examining the main effect of diagnosis across all hormone conditions revealed no regions in which resting rCBF differed between controls and women with PMDD. In contrast, the main effect of hormone condition across all participants was seen in several foci in brain regions detailed in Supplementary Table 1. These included the orbitofrontal cortex and medial prefrontal cortex (PFC), subgenual cingulate cortex (SGCC), inferior and superior temporal gyri, primary motor cortex, and supramarginal gyrus. Post-hoc pairwise analyses testing for between-hormone effects within these brain regions revealed that in the inferior and superior temporal gyri, resting rCBF was significantly greater in the Lupron-alone condition compared with both estradiol and progesterone conditions, and that rCBF was significantly greater during estradiol add-back compared with the progesterone add-back. In addition, in the orbitofrontal cortex, medial PFC, and SGCC, rCBF was significantly greater during both estradiol and Lupron-alone compared with progesterone add-back. Finally, in the primary motor cortex and supramarginal gyrus, resting rCBF was increased in the estradiol condition compared with both the progesterone and Lupron-alone conditions (*P*’s ≤ 0.04 for all post-hoc comparisons).

### Diagnosis-by-hormone interactions in resting rCBF

**Fig. 2:**
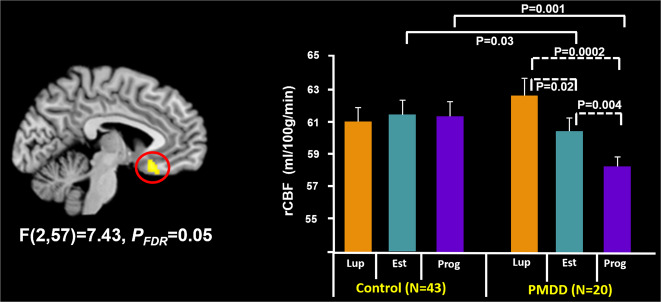
Statistical parametric map showing a diagnosis-by-hormone interaction in the subgenual cingulate (SGCC) rCBF and post-hoc analyses. Left: Statistical parametric map showing voxels with a diagnosis-by-hormone interaction (*P*_FDR_ ≤ 0.05) in the subgenual cingulate (BA25, peak voxel MNI coordinates: −6, 20, −6). Right: *Post-hoc* analyses revealed that in healthy controls, there were no hormone-related rCBF differences in this region (*P*’s ≥ 0.3). In contrast, in women with PMDD, we observed differences in rCBF across hormone states (all comparisons *P* ≤ 0.02), suggesting differential modulation of this region by ovarian steroids in PMDD. In addition, compared with controls, women with PMDD had similar levels of resting SGCC rCBF during Lupron alone (when PMDD symptoms remit) but had decreased rCBF during both estradiol (*P* = 0.03) and progesterone (*P* = 0.001) conditions (when symptoms are likely to recur). Solid line brackets show comparisons between diagnosis and dashed line brackets show comparisons across hormone conditions in women with PMDD. Est estradiol, Lup lupron, Prog progesterone.

In the study’s key analysis, basal, resting rCBF in a single locus, the subgenual cingulate (SGCC, MNI *x*, *y*, *z* = −6, 20, −6; *P*_FDR_ = 0.05), demonstrated an interaction between diagnosis and hormone condition. Post-hoc analysis in this region showed that compared with controls, women with PMDD had similar levels of resting SGCC rCBF during Lupron-alone condition (when PMDD symptoms remit) but had decreased rCBF during both estradiol (*P* = 0.03) and progesterone (*P* = 0.001) conditions (when symptoms are likely to recur). In addition, while control women showed no changes in resting rCBF across the three hormone conditions, those with PMDD showed decreased resting rCBF during both estradiol (*P* = 0.02) and progesterone add-back conditions (*P* = 0.0002) compared with Lupron alone. The most robust SGCC rCBF differences were identified during progesterone add-back, both when compared between groups and when compared with rCBF during hypogonadism (i.e., the Lupron-alone condition) in women with PMDD (Fig. 2), consistent with observations of recurrence of PMDD symptoms during this hormonal condition^5^. Finally, in women with PMDD, there were no significant correlations between SGCC resting rCBF, PMTS ratings, and plasma hormone levels (all comparisons *P* ≥ 0.05).

### Exploratory analysis of resting rCBF correlation with ESC/E(Z) complex expression

**Fig. 3:**
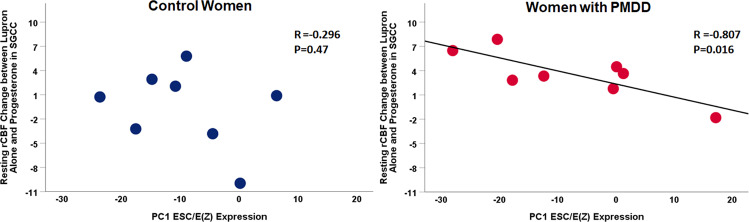
Relationship between ESC/E(Z) gene expression and the difference in SGCC resting rCBF activity between Lupron alone and progesterone add-back conditions. Pearson correlations between the first principle component of baseline LCL ESC/E(Z) gene expression and the difference in SGCC resting rCBF activity between the progesterone add-back and Lupron-alone conditions in healthy control women (left) and women with PMDD (right). In women with PMDD, the first principle component of the ESC/E(Z) expression significantly correlated with the change in resting rCBF between Lupron-alone and progesterone add-back conditions (Pearson *r* = −0.807, *P* = 0.016), whereas no correlations were observed in healthy controls (Pearson *r* = −0.296, *P* = 0.476).

Eight healthy control women (age = mean(SD) 36.2 ± 6.0 years) and eight women with PMDD (41.5 ± 6.4 years; *t*(15) = −1.7, *P* = 0.1) had both LCLs and resting rCBF data. Because of the observed SGCC resting rCBF differences between the Lupron alone condition and the estradiol and progesterone replacement conditions in the PMDD group, values for rCBF change between progesterone and Lupron-alone conditions, as well as between estradiol and Lupron-alone conditions were used in this correlational analysis. In the PMDD group, the first principle component of the ESC/E(Z) expression significantly correlated with resting rCBF change between progesterone add-back and Lupron-alone conditions (Pearson *r* = −0.807, *P* = 0.016), whereas no correlations were observed in healthy controls (Pearson *r* = −0.296, *P* = 0.476; Fig. 3). In addition, there were no significant correlations between gene expression and resting rCBF change between Lupron-alone and estradiol conditions in either PMDD (Pearson *r* = −0.461, *P* = 0.251) or healthy control women (Pearson *r* = −0.238, *P* = 0.570).

## Discussion

We tested for neural substrates associated with the differential behavioral and cellular response to ovarian hormones in PMDD. In women with PMDD but not in controls, exposure to physiological levels of estradiol and progesterone impacted resting rCBF in the SGCC, a region involved in affective regulation and in the pathophysiology of affective disorders^27^. In particular, while basal, resting rCBF was comparable in the two groups when no ovarian hormones were present (i.e., during the Lupron-alone condition), in the PMDD group, resting rCBF was decreased compared both to controls and to the Lupron-alone condition during both estradiol and progesterone add-back (when PMDD symptoms are at risk of recurring^5^). Moreover, in PMDD, the change in SGCC resting rCBF between the Lupron-alone and progesterone conditions correlated with gene expression in the ESC/E(Z) complex. These results add to an emerging clinical, neurobiological, and cellular framework for understanding the role of ovarian steroids in brain function in women with PMDD.

The apparent specificity of the rCBF findings to the SGCC is of particular interest. This brain region is involved in the regulation of affect, and both structural and functional abnormalities in the SGCC have been documented in mood disorders, particularly in major depressive disorder (for reviews, see refs. ^27,28^). As such, this brain region has become a key target for neuromodulation (i.e., deep brain stimulation) in treatment-resistant depression^29,30^. While the SGCC holds primacy in the present rCBF results, our small sample size limits our ability to rule out the possibility that other brain regions may show similar hormone-related and diagnosis-specific changes. Likewise, we cannot disambiguate the possibilities that the SGCC rCBF findings reflect a direct effect of ovarian hormones that manifests in a negative affective state, or that the differential activity is secondary to the altered mood state in women with PMDD. These caveats notwithstanding, our findings of ovarian steroid-related alterations in basal, resting SGCC activity in PMDD are not only consistent with the existing literature documenting the importance of the SGCC in regulating affective state and in affective disorders but are also biologically plausible. For example, this brain region is reported to contain high levels of serotonin receptor and transporter^31^, which is of potential importance to our findings given the specificity of the therapeutic response of PMDD to serotonergic agents (including SSRIs and SNRIs) compared with non-serotonergic tricyclic antidepressants^1^. In addition, although few studies have tested for the presence of estrogen or progesterone receptors in the human SGCC, studies in animals have reported the presence of progesterone receptor mRNA^32^, as well as estrogen receptor mRNA and protein^33,34^ in the cingulate gyrus and frontal cortex. Indeed, a recent whole-transcriptome sequencing study in humans demonstrated the presence of progesterone receptor membrane component 2 (PGRMC2) and nuclear progesterone receptor gene expressions within the SGCC^35^, suggesting the possibility of direct effects of progesterone on SGCC neurons.

Importantly, the SGCC also expresses several genes in the ESC/E(Z) complex^35^ (an ovarian steroid-regulated gene-silencing complex^36–38^), and we have previously reported intrinsic cellular differences in this complex in LCLs from women with PMDD compared with healthy women^17^. Building on this work, we searched for preliminary evidence of gene-regulatory underpinnings of the neurophysiological (rCBF) findings. Albeit in a small sample size, we found a relationship between gene expression in the ESC/E(Z) complex and the effects of ovarian steroids on SGCC rCBF—only in women with PMDD and only with the rCBF change between the Lupron-alone and progesterone conditions. Interestingly, among the rCBF results, the change between Lupron-alone and progesterone conditions in PMDD was the most robust. Consistent with the relevance of this finding, we recently demonstrated that preventing the luteal phase increase of progesterone neurosteroid metabolite allopregnanolone prevented the onset of PMDD symptoms, thus implicating the roles of progesterone and its metabolite in triggering PMDD^39^. Thus, the neural substrate (i.e., SGCC resting rCBF) underlying the PMDD behavioral phenotype may be mediated by intrinsic cellular differences in the ESC/E(Z) complex. While alterations in SGCC rCBF and ESC/E(Z) gene expression could be independent events, it is possible that altered expression of ESC/E(Z) complex genes in the SGCC directly impacts SGCC neurophysiology and, therefore, contributes to the ovarian steroid-induced alterations in rCBF we observed in PMDD. Alternatively, the physiological effects associated with progesterone in the SGCC could induce increased expression of ESC/E(Z) complex genes. Although disambiguating these mechanisms will require further investigation, our preliminary findings suggest an important relationship between intrinsic cellular function and the neurophysiological response to ovarian steroids in the pathophysiology of PMDD.

In conclusion, our study not only identified changes in basal, resting brain function in women with PMDD, we did so under controlled hormone conditions in which the exposure to ovarian steroids (i.e., dose and duration) was standardized across women with PMDD and controls. We, thus, observed differences between PMDD and controls that were not confounded by differences in circulating levels of estradiol and progesterone across the menstrual cycle. Moreover, our rCBF findings, together with the RNA-seq data, may offer a perspective on PMDD that integrates behavioral, neural circuit, and cellular mechanisms. Such multi-level formulations could provide new targets for, and approaches to, future therapeutic interventions.

## Supplementary information

Supplementary Table 1: Brain areas showing main effects of hormone conditions
